# Integrating Quality Tools and Methods to Analyze and Improve a Hospital Sterilization Process

**DOI:** 10.3390/healthcare9050544

**Published:** 2021-05-07

**Authors:** Amira Kammoun, Wafik Hachicha, Awad M. Aljuaid

**Affiliations:** 1 OLID Research Laboratory, Higher Institute of Industrial Management Sfax, University of Sfax, Technopolis of Sfax 3021, Tunisia; emira.kammoun@gmail.com; 2 Department of Industrial Engineering, College of Engineering, Taif University, P.O. Box 11099, Taif 21944, Saudi Arabia; amjuaid@tu.edu.sa

**Keywords:** failure modes and effects analysis (FMEA), quality function deployment (QFD), cause and effect analysis (CEA), structured analysis and design technique (SADT), hospital sterilization process, reusable medical devices (RMD), process improvement

## Abstract

Healthcare facilities are facing major issues and challenges. Hospitals continuously search approaches to improve operations quality, optimize performance, and minimize costs. Specifically, an efficient hospital sterilization process (HSP) allows reusable medical devices (RMDs) to be more quickly available for healthcare activities. In this context, this paper describes an integrated approach developed to analyze HSP and to identify the most critical improvement actions. This proposed approach integrates four quality tools and techniques. Firstly, a structured analysis and design technique (SADT) methodology is applied to describe HSP as a hierarchy of activities and functions. Secondly, the failure modes and effects analysis (FMEA) method is used as a risk assessment step to determine which activity processes need careful attention. Thirdly, a cause–effect analysis technique is used as a tool to help identify all the possible improvement actions. Finally, priority improvement actions are proposed using the quality function deployment (QFD) method. To validate the proposed approach, a real sterilization process used at the maternity services of Hedi-Cheker Hospital in the governorate of Sfax, Tunisia, was fully studied. For this specific HSP, the proposed approach results showed that the two most critical activities were (1) improving the coordination between the sterilization service and the surgery block and (2) minimizing the average duration of the sterilization process to ensure the availability of RMDs in time.

## 1. Introduction

### 1.1. Research Motivation

All healthcare systems are confronted with the triad of performance: improving quality of care, customer satisfaction, and cost reduction. It follows that it is necessary to reduce the operating costs through evaluation and improvement of internal hospital processes. Due to the growing demands for quality improvement and the increasing pressure from regulatory agencies, issues regarding how to provide high-quality hospital services and improve patient satisfaction have caught the attention of hospital administrators [1,2]. Specifically, an efficient hospital sterilization process (HSP) ensures that reusable medical devices (RMDs) are more rapidly available for healthcare activities. RMDs are an important and growing aspect of healthcare provision; their complexity is increasing to meet established and emerging patient needs. The HSP plays a vital role in the provision of safe RMDs [3]. As these RMDs come in contact with the human body, it is essential to attain sterility, which, in simple terms, means the absence of all living organisms. If contaminated instruments are not cleaned and sterilized appropriately, they may cause serious infections in patients. Similarly, if poor logistics management leads to instruments missing when they are needed, patient lives may be endangered [4]. Notably, these RMDs are generally sterilized in centralized sterilization facilities.

This research started from two major ascertainments. The first is about the applicability of quality methods in healthcare. The concepts of quality, quality management, and total quality management (TQM) have a long history and are commonly applied in the industry. The management of quality and its control are not as advanced in healthcare as in the industry [5]. Likewise, TQM promises much for service industries, yet it has been little used in healthcare [6]. According to Komashie et al. [7], this is due to several reasons: (1) the large differences between the two sectors in terms of concerns for quality and the type of processes and outputs involved, (2) the fact that quality researchers have differing views toward the best approaches, and (3) the fact that consumers of healthcare have little knowledge about their needs, unlike in those of industries. There is therefore little historical evidence of healthcare consumers demanding any level of quality. Consequently, the quality of healthcare has been a much-debated issue all over the world. It seems that in finding a definition, the methods of evaluation, quality monitoring, and quality improvement should be key issues for both researchers and healthcare professionals. The second ascertainment is about the improvement in HSP. Over the last few decades, many researchers and practitioners have conducted studies focused on the issue of HSP of RMDs. These previous studies can be grouped into two categories. The first includes purely technical and microbiologic technological studies of sterilization process activities [3,8,9]. The second category concerns the evaluation of and the improvement in HPS using operations research and management approaches. This second category can be also classified in the literature into many subcategories, including: (1) RMD inventory management, such as in Nilsen [10], Ahmadi et al. [11], etc.; (2) optimization of the RMD flow in hospitals using mathematical programming or cost analysis approaches, such as in Van de Klundert et al. [4], Fineman and Kapadia [12], Johnson [13,14], Reymondon et al. [15], Ozturk et al. [16], etc.; (3) simulation of RMD flow, such as in Di Mascolo and Gouin [17], Hachicha et al. [18], etc.; and (4) evaluation of and performance improvement in HSP using engineering management tools, such as in Weinstein et al. [19] and, recently, in Figliatto et al. [20]. For instance, Weinstein et al. [19] proposed that failure mode and effects analysis (FMEA) be applied to HSPs to examine potential failure modes and their causes, as well as to score the severity and other factors for each failure mode cause. Figliatto et al. [20] proposed a three-phase method based on lean healthcare principles, cluster analysis, and kaizen groups of experts to ration surgical trays, thus reducing sterilization processing costs.

As observed from the literature review above, an approach has not yet been proposed to study HSPs by developing an integrated methodology based on quality methods in the literature. To the best of our knowledge, this is the first study applying an integrated methodology based on FMEA and quality function deployment (QFD) to HSP improvement. Consequently, the proposed study can be classified as belonging to the fourth subcategory.

The improvement in HSP has not received the same degree of attention as other hospital processes such as admission processes, emergency room operations, patient flow, etc. There are many quality–method integrations proposed in the literature. In the following, only recent papers are presented. For instance, Harikumar and Saleeshya [21] presented a case study dealing with risk identification, measurement, assessment, mitigation, and control of risks in hospitals. They applied the FMEA and QFD methods for risk quantification and assessment. By using these techniques, the critical risk agents that created risk events in the hospital were identified. Altuntas and Kansu [22] proposed an integrated approach based on service quality measurement and QFD and FMEA methods to improve the quality of service in a public hospital in Turkey. Pourmadadkar et al. [23] presented an integrated approach using FMEA, multiple-criteria decision making, and QFD techniques for risk assessment and service quality enhancement in coronary artery bypass grafting as a treatment for cardiovascular diseases.

### 1.2. Literature Overview

The rapid growth and the dramatic changes in the hospital industry are challenging healthcare managers to find alternative methods to preserve the quality of hospital services. Improving the capacity to produce acceptable results, ensuring the safety of patients and personnel, and improving service quality have become important objectives for any healthcare system. Therefore, the main concern of healthcare supply chain management is its performance. In most studies, the concept of performance used quality as its dimension [24]. For this reason, healthcare managers have turned to total quality management (TQM) [25], since implementing TQM tools and techniques enables organizations to capture and re-design their services to meet customers’ requirements, help with creative thinking and problem solving, and produce continuous improvement in performance.

The following literature overview only focuses on the use of quality methods to improve hospital processes. Notably, the main TQM tools are FMEA and QFD [26].

#### 1.2.1. Failure Mode and Effects Analysis (FMEA)

FMEA is a proactive risk assessment tool used to identify potential vulnerabilities in complex, high-risk processes and to generate remedial actions to counteract them before they result in adverse events. It is generally acknowledged to be a useful tool available to health professionals for assessing and improving healthcare processes [27,28,29]. It is a good systematic technique that prospectively identifies, evaluates, prioritizes, and eliminates potential failure modes and effects to improve the safety, reliability, and quality of healthcare processes [30,31,32,33,34].

Day et al. [35] used FMEA analysis to identify strategies that reduce risks and improve patient safety during the registration of trauma patients and subsequent electronic data linkage. The authors recommended the application of a method of evaluation to other healthcare processes. Capunzo et al. [36] experimented with FMEA application in a clinical laboratory and demonstrated it can be applied to the processes in a clinical laboratory and offers a high potential for improvement. Chiozza and Ponzetti [37] applied the FMEA to a testing process and reviewed data available on the application to laboratory medicine. Najafpour et al. [38] conducted a risk evaluation of a blood transfusion process in a general teaching hospital using FMEA. Additionally, Malfará et al. [39] adopted FMEA to detect drug-related hazards within the pediatric ICU of a tertiary university hospital, and the critical failure modes were reduced by clinical pharmacist interventions.

The widespread use of FMEA in different sectors demonstrates its ease of implementation and its adaptability. Despite this, Chiozza and Ponzetti [37] defined some weaknesses of this tool such as the uncertainty of determining risk factors (O, G, and D) and the criticality value making the final decision making subjective. Therefore, the most proposed improvements focused in particular on the robustness of decision making and the coupling of FMEA with other tools [40]. Liu et al. [41] provided a comprehensive review for the period 1998–2018 of the FMEA studies using multiple-criteria decision-making approaches for evaluation and prioritization of failure modes.

#### 1.2.2. Structured Analysis and Design Technique (SADT)

The SADT method has frequently been used in manufacturing systems. It is based on two basic constructs: function box and arrows. The function box represents activities, processes, and transformations; the arrows represent data and objects related to the functions. A previous study showed that any kind of system can be modeled using SADT [42]. In the healthcare sector, the SADT method has been used to model several processes in different hospital departments such as radiation oncology [43], surgical processes, [44] and emergency [45].

Bevilacqua et al. [46] developed a systemic approach to detect waste and errors and to suggest organizational and/or technological solutions for continuous improvement in the pharmacy department. The proposed framework integrates the structured analysis and design technique (SADT) and FMEA.

#### 1.2.3. Quality Function Deployment (QFD)

The primary functions of QFD are quality management, product development, and customer needs analysis. Nowadays, QFD functions have expanded to various fields such as decision making, engineering, management, costing, healthcare, etc. QFD is a planning methodology used to improve products, services, and their associated processes by ensuring that the voice of the customer (VOC) is effectively deployed through specified and prioritized products or service. It is also a flexible tool that can be fashioned to be effective in a wide range of applications and for many types of organizations with many commonly known benefits [47,48,49]. Gremyr and Raharjo [47] conducted a literature review on the use of QFD, focusing on its possibilities and antecedents. Carnevalli and Miguel [50] highlighted QFD ability to adapt to various research methods from modeling to theoretical–conceptual and action–experimental methods. In the literature, four potential applications of QFD were suggested: to achieve to a better understanding of customers’ needs and wants, to help identify opportunities for process improvement that play key roles in meeting customers’ most important needs, to facilitate an effective system thinking approach, and to provide better communication and a more transparent process through performance measurement. QFD has not been widely applied in healthcare management, although research in this area is increasing. It requires a slightly different approach with respect to applications in other service industries [26].

Said et al. [51] used the QFD method to provide quality inpatient service based on patients’ expectations. Debata et al. [52] proposed an integrated approach based on QFD and interpretative structural modeling to achieve the highest levels of medical tourists’ satisfaction in India.

Recent attempts to apply the QFD method to the healthcare sector concentrated on customers’ needs and how to engineer the process. In particular, authors found that the patient was not necessarily the only customer and that it was better to consider stakeholders or strategically related interested groups, such as reference groups (consultant physicians), local and national governmental authorities, taxpayer and/or insurance companies, and hospital management and staff. Stamatis [53] defined the customer as the person or unit receiving the output of a process or a system. The customer may be an immediate, an intermediate, or an ultimate customer and may be a person or a process.

### 1.3. Objective and Contributions of the Study

This paper has two main contributions. The first concerns the development of an integrated approach based on four quality tools and methods. Firstly, SADT methodology is applied for describing the HSP as a hierarchy of activities and functions. Secondly, FMEA is used as an assessment risk step to determine which activity processes need careful attention. Thirdly, cause and effect analysis (CEA) is applied as a tool to help identify all the possible improvement actions. Finally, priority improvement actions are proposed using QFD. To validate the proposed approach, a real sterilization process used in a university hospital in Tunisia [18] was fully studied. The second contribution concerns the application of the proposed integrated approach to a sterilization process, which has not yet been conducted in the literature. The proposed approach deals with the application of two main TQM tools (QFD and FMEA) in healthcare quality management.

## 2. Materials and Methods

### 2.1. The Proposed Approach

**Figure 1:**
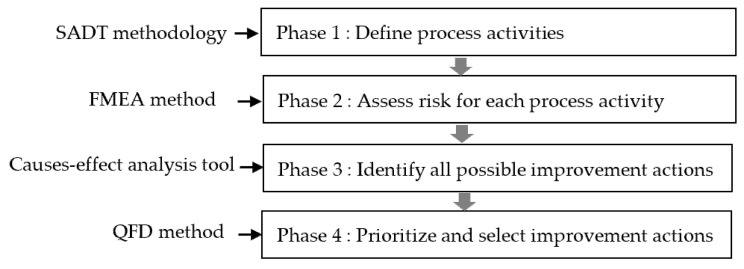
Flowchart of the proposed approach.

The proposed approach integrates four quality tools and techniques. Firstly, the SADT is applied for describing the HSP as a hierarchy of activities and functions. Secondly, FMEA is used as an assessment risk step to determine which activity processes need careful attention. Thirdly, CEA is used as a tool to help identify all the possible process actions that can be improved. Finally, priority improvement actions are proposed using QFD. Figure 1 presents the flowchart of the conceptual framework of the proposed approach.

To overcome the different challenges regarding improvement in the healthcare supply chain, the decision maker needs to identify the functional aspects of a process and consider nurses’ expectations. Therefore, the improvement process requires the evaluation of the actual state of activity and the definition of an improvement plan. However, failures analysis must be considered in the improvement decision process. For this, FMEA was used to determine which activity processes needed more attention. Then, to determine possible improvement actions, we considered the operator requirements using cause–effect analysis.

The QFD method allows consideration of the improvement operator requirements and the identification of the importance of each requirement in the decision. Therefore, an improvement process design was proposed.

Through this research work, we constructed a new combination of FMEA and QFD to identify priority improvements actions. Both techniques require a systematic process of what/how and cause/effect. For this, the SADT was integrated in our approach to define the different activities and steps of the process studied (what), and the cause–effect analysis was applied to define improvements actions according to the CEA (also called the 5-M diagram) to help the organization find viable management solutions. It is a real diagnostic tool allowing a global and interdependent view of the problem.

### 2.2. The Case Study

For the case study, we selected the sterilization process used at the maternity service of Hedi-Cheker Hospital located in the governorate of Sfax, Tunisia. This maternity service performs approximately 15,000 surgical procedures per year [18]. This high volume is accompanied by relatively weak systems for tracking equipment and instruments. Often, this results in delays in procedures because RMDs are not available. Each delay generated can cause significant delays in the subsequent procedures. The sterilization process contributes indirectly to the management of patient services. Its purpose is to provide secure and quality patient care services, being a key service in high-quality patient care.

The HSP usually includes, in addition to the sterilization phase, decontamination, collection, wash, storage packaging, and distribution of the various RMDs. Doctors, surgeons, and nurses were considered as the direct customers of this process. In the surgery block, to minimize the operating risks, the expectations of the operating room staff and the analysis of the actual flows of reusable medical device production should be carefully considered to improve the efficacy and profitability of sterilization.

The studied maternity services occupied two floors, six operating rooms, and three surgical departments: gynecology, obstetrics, and neonatology. Most of the time, the HSP works 24 h per day [18]. Each department carries out numerous surgical procedures with a large variety of RMDs consisting of instruments devices, clothing, small plastic equipment, etc. At the end of every surgical operation, each RMD undergoes an accurate HSP before being re-used.

## 3. Results

### 3.1. Phase 1: Define Process Activities

**Figure 2:**
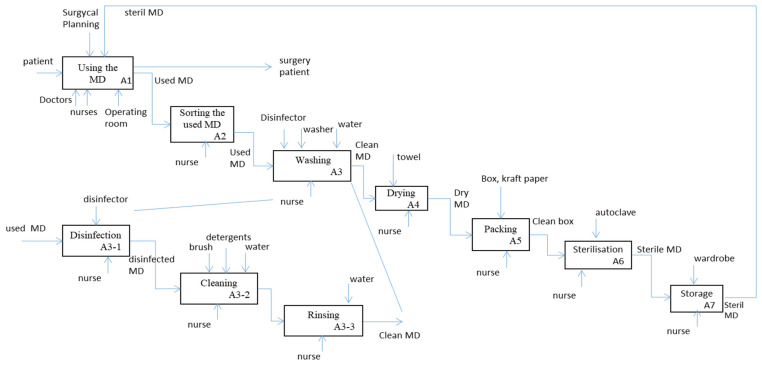
The sterilization process studied through an IDEF0 diagram.

To define the sterilization process, we used SADT. The activities and the process design containing all the information process phases are presented. This step defines the customer requirement (CR) of the QFD method. To identify all the activities occurring within the sterilization service, the actual process was described in detail using the SADT graphics. All the sterilization steps start when the surgery operation finishes. Surgeons and nurses were considered as the users of the RMDs (customers of the hospital sterilization service), and different steps of the hospital sterilization were defined by the sterilization standard. The different activities are summarized in Figure 2.

The process reported in the IDEF0 diagram in Figure 2 describes what actually happens in daily practice. It is possible to see that the wash step was a manual activity, which increases the length of the process, especially when the nurses are busy.

### 3.2. Phase 2: Assess Risk for Each Process Activity

Process risks were assessed using a risk analysis based on FMEA, which describes the different failure modes, classifies the risks sources, determines the causes and effects of errors, and proposes some possible corrective measures. Therefore, FMEA was used to identify risk, its current location, and its effects. Failure may be due to human error, equipment problems, communication difficulties, and missing personnel or materials, or any other cause that might disrupt the material flow and the safety of the process. In the absence of sufficient quality control mechanisms, failure occurrences increase.

After identifying all potential failures, the possible causes and effects were discussed with the service staff. Then, for each failure mode, severity, occurrence, and detection were defined by the nurses of the surgical blocks. Table 1 shows the conversion of each parameter situation into a numerical value.

The severity, occurrence, and detection levels were fixed for each failure mode, and we calculated the risk priority number (RPN). RPN is calculated as:

RPN = severity (S) × occurrence (O) × detection (D).(1)

As can be seen from Equation (1), there are three indicators, severity (S), occurrence (O), and detection (D). Each indicator should be measured by an integer value between 1 and 5. To define the different levels of severity, occurrence, and detection, a brainstorming process was conducted with the hospital managers, and it was concluded that it was easy to detect the defined failure mode. Thus, the applied detection level of each failure mode was considered equal to 1 (D = 1) for all failure modes. The RPN depends only on occurrence and severity. Therefore, all possible remaining situations are presented in Table 2. Additionally, three situations are considered in Table 2: the green color indicates an acceptable risk, orange indicates an unwanted risk, and red indicates an unacceptable risk.

To identify the major critical activities, a brainstorming process was conducted with the hospital managers. Table 3 shows the obtained FMEA table. For each HSP activity, the failure mode (column 2 of Table 3) was defined. Once the potential failures were identified, the potential effects (column 3 of Table 3) and all possible causes (column 4) were determined. Historical records stored in databases were used to determine the occurrence (O) of each failure mode. However, hospital managers evaluated the severity (S) based on their experience. Each point presented in this FMEA table was established following various discussions with the managers, doctors, and nurses. The severity, occurrence, and detectability for each failure mode was estimated based on the levels indicated in Table 1. The RPN was calculated using Equation (1).

Table 3 indicates that the RPN ranged from 12 to 25. The majority of the studied risks were unacceptable (RPN > 15). There were many critical risks such as the decontamination time not being sufficient during the disinfection phase, lack of control during the main sterilization activities, and poor RMDs transport conditions. The packaging phase was assigned the lowest RPN of 12. All HSP activities were classified according to their RPN in descending order. However, one main disadvantage of FMEA is that the relationship between different failure components is disregarded [54]. During a brainstorming meeting, hospital managers insisted on describing the impact of each activity in addition to the RPN scores. Therefore, the final ranking is indicated in the QFD matrix in Table 4 based on the RPN scores and the priority of each step. For example, the disinfection activity must occur before transportation. The hospital managers considered RMDs disinfection as being more important than RMDs transport.

### 3.3. Phase 3: Identify All Possible Improvement Actions

The nurses’ requirements were classified using a CEA diagram. CEA can help an organization to find viable management solutions. The purpose of HSP is to provide other departments with sterile RMDs for their technical, surgical, and care activities in optimal conditions of safety and cost. The possible design requirements were defined through an analysis of the responses provided by nurses about their actual experiences with the hospital sterilization service. The possible design requirements (DRs) and their improvement actions are described in Table 5.

### 3.4. Phase 4: Prioritize the Design Requirements and Improvement Actions

In the literature, many studies have applied QFD [55]; however, the minimum QFD model contains at least the requirements and problems (the whats) and their relative importance (why), technical measures or design requirements (the hows), their relationships with the whats, and the correlation between the hows.

In this study, the QFD method was used to determine the priority improvement solution considering the views of the nurses and operational personnel in the sterilization process. The criticality values according to the FMEA analysis were used to determine the priority level of the process activities as customer requirements (CRs). Then, a new configuration of the QFD methodology, using the FMEA rating and the causes–effects diagram categories, was constructed.

Based on the QFD model, customer needs, existing problems of the organization (process activities), and the HSP design requirements were derived. Then, an analysis was conducted to link the customers’ needs and DRs and map nurses’ and standards requirements into a process. We aimed to calculate the weights of the design requirements (DRs) of the sterilization process, so to meet surgeons’, nurses’, and normative requirements.

The element CRi (what) represents the HSP activities. The element DRj (how) is the improvement category. The various steps involved in the QFD methodology are presented below [49,56]:

- Identify the customer requirements (what) that represent the sterilization process steps. The sterilization process design was identified through direct observation and interviews with nurses and instrumentalists of the surgical blocks. Items were entered into the house of quality (HOQ) as a voice of the customer (VOC) inputs.
- Prioritize the CRs using the FMEA rating and a number that reflects the importance of the demand using a one-to-five scale. For sterilization and disinfection activities, which were ranked 1 and 2, respectively, according to FMEA, a weight of 5 was attributed to these activities. For the transport, storage, and RMD sorting activities, the attributed weight was based on the fact that their FMEA ranking ranged between 3 and 5. Finally, the washing and packing activities, which had the lowest FMEA ranking, were attributed a weight of 3. Each activity weight is indicated in the third column of Table 4.
- Determine the categories requiring improvement using indicators that represent the DRs using surveys and exploratory factor analysis. A questionnaire survey was administered to determine the categories requiring improvement based on nurses’ and normative requirements (good pharmaceutical practice).
- To apply the QFD method, the customer should define a relative importance (*W_i_*) of each customer’s requirements (*CR_i_*). In our case study, the *W_i_* was defined by healthcare managers for each HSP activity based on the good pharmaceutical practice of the HSP (technical importance), and the risk priority of each HSP activity was defined by the FMEAC analysis. The *W_i_* constitutes the link between FMEA and the QFD method. The RPN calculated by the FMEA for each activity of the process guide and help the decision maker to determine the *W_i_* of each *CR_i_*.
- Determine the what–how relationships, which represent the degree to which *CR_i_* is met by *DR_j_*. These relationships can be evaluated according to a rating scale. In the literature related to QFD [49], many methods and a set of rating scales to facilitate gathering and displaying information are available. A three-point ordinal scale (weak, medium, and strong) can be used to establish relationships between *DR_j_* and *CR_i_*. This scale considers a further point: the absence of relationship. Ratings obtained with this scale are usually scalarized with numerical series 0, 1, 3, and 9 or 0, 1, 3, and 5 [56]. There is no competition between the rating scales, but the use of one provides an evaluation of the relationships to classify them and then to detect the avenues of HSP improvement. In this study, a 0, 1, 3, 9 rating scale was used. The interrelations are typically defined as strong (9), moderate (3), weak (1), and none (0). In other words, for each activity *i*, each *DR_ij_* is estimated using four possibilities: 9, 3, 1, or 0.
- Calculate the individual *DR_j_* indices for each *DR* using Equation (2). The values are shown in the last row of Table 5. For example, using Equation (2), the *DR* index of the equipment was equal to 4 × 3 + 5 × 0 + 3 × 3 + … 4 × 9 = 165.
  (2) $${DR}_{j}\;index=\overset{}{\underset{All\;activities\;i}{\sum }}W_{i}\times {DR}_{ij}$$
- Classify the *DR_j_* according to its index value. Derived from the results of the QFD matrix (Table 4), the most important *DR* for the improvement in the HSP was the management action category, which had an index value of 222. Using the details of the Management category in Table 5, we concluded that the coordination between sterilization service and the block, the average duration of the sterilization process, and the availability of sterile RMDs at a time were the most important factors for improving the current HSP. The second and third most important DRs were personnel category (value of 204) and method category (value of 192). The equipment category (value of 165) was ranked as the fourth important improvement action. Verified by the hospital management, the results were regarded as practical and informative.

## 4. Conclusions

In this study, we developed and applied an integrated approach to help healthcare decision makers to evaluate actual hospital processes and define priority improvement actions. This approach reveals the most important and urgent actions that will help to achieve the highest levels of process performance. This proposed approach integrates four quality tools and techniques. Firstly, the SADT was applied for describing the HSP as a hierarchy of activities and functions. Secondly, FMEA was used as an assessment risk step to determine which activity processes needed careful attention. Thirdly, CEA was used as a tool to help identify all the possible improvement actions. Finally, priority improvement actions were proposed using the QFD method.

The main benefit of the proposed approach is that it provides an overall evaluation of the service system by considering HSP requirements, service design, and possible failures. A real-life case study in a large public hospital located in Tunisia was conducted to demonstrate how the proposed innovative approach works in practice. For this specific case, the proposed approach results identified the two most critical activities: (1) improving the coordination between the sterilization service and the surgery block, and (2) minimizing the average length of the sterilization process to ensure the availability of RMDs in time. The results obtained from this case study reveal that decision makers can straightforwardly use the proposed innovative approach for service quality improvement.

The underlying premise of the research is sound and important. Applications of innovative quality management approaches and paradigms are always needed to improve the quality of key hospital processes, especially in developing countries that are grappling with resource scarcity and suboptimal personnel training. To ensure process improvement success, there are three things HSP managers need to know: first, the obtained results of this study may not be complete or perfect, but managers, according to their implication, will have implemented a positive change. Secondly, it is wise to actively involve the hospital employees and customers in improvement efforts. Finally, managers will be prepared for natural employees’ resistance to change.

For academic continuity, this paper provides useful guidelines for effective risk management in hospitals and shows how quality methods can be integrated into the hospital system. Some future works are envisaged such as, firstly, the application of the proposed approach to other HSPs and, secondly, the addition of the waste analysis component to the proposed approach to obtain a more effective improvement process.

## Figures and Tables

**Table 1:** Likelihood of occurrence, severity, and detectability parameters.

Value	Occurrence (O)	Severity (S)	Detectability (D)
1	Less than once a year	No harm, no increased	Observable
2	Less than once a month	Non-permanent minor harm or increased length of stay	Effective detection
3	Less than once a week	Non-permanent major harm or permanent minor harm	Low detection
4	Less than once a day	Permanent major harm	Rare detection
5	Once a day or more	Death	Cannot detect

**Table 2:** Matrix frequency and severity.

Occurrence/Severity	1	2	3	4	5
5	5 Un-wanted	10 Un-wanted	15 Un-acceptable	20 Un-acceptable	25 Un-acceptable
4	4 Acceptable	8 Un-wanted	12 Un-wanted	16 Un-acceptable	20 Un-acceptable
3	3 Acceptable	6 Acceptable	9 Un-wanted	12 Un-wanted	15 Un-acceptable
2	2 Wanted	4 Acceptable	6 Acceptable	8 Un-wanted	10 Un-wanted
1	1 Wanted	2 Wanted	3 Acceptable	4 Acceptable	5 Un-wanted

The white color indicates a wanted risk, the green color indicates an acceptable risk, the orange color indicates an unwanted risk, and the red color indicates an unacceptable risk.

**Table 3:** FMEA table of the hospital sterilization process.

Activity	Failure Mode	Effect	Cause	S	O	D	RPN
Reception and sorting soiled medical devices	Queue of soiled RMDs	Damage to the RMDs	Nurses are busy	4	4	1	16
Disinfection	Decontamination time is not respected	Medical dispositive was poorly disinfected	Staff are not trained	5	5	1	25
Washing	Security measures are not followed	Risk of nurse’s infection	Lack of personnel safety tools	4	4	1	16
Drying	Lack of a special drying tool	Medical device poorly dried	Using a towel to dry the medical device	3	5	1	15
Packaging	Queue of packaged, clean medical devices	Late delivery of the RMDs	Insufficient sterilization equipment	3	4	1	12
Sterilization	Lack of control	Service is badly organized and does not meet pharmaceutical standards	No sterilization activity manager	5	5	1	25
	Heterogeneous load of the autoclave	Some medical devices are poorly sterilized	Lack of awareness and control	5	4	1	20
	Autoclaves frequently out of order	Disruption of activities and surgical schedule	Equipment amortized and absence of preventive maintenance	4	4	1	16
	Overload of the autoclave	Boxes are wet and badly sterilized	Lack of autoclave baskets	5	4	1	20
Storage	Sterile RMDs poorly stored	Medical device risk due to no longer being sterilized	Absence of adequate storage sites	4	5	1	20
Transport	Poor transport of the RMDs	Damage to the boxes	Lack of transport trolleys	5	5	1	25

**Table 4:** QFD matrix (house of quality).

What: *CR_i_*	FMEA Ranking	*W_i_*	How: *DR_j_*						
			Equipment	Material	Method	Environment	Personnel	Measure	Management
RMD sorting	5	4	3	0	9	0	3	0	9
Disinfection	2	5	0	9	9	9	9	0	9
Washing	6	3	3	3	3	9	9	3	9
Packing	7	3	9	0	3	9	9	3	9
Sterilization	1	5	9	3	9	9	9	9	3
Storage	4	4	9	0	3	0	3	3	9
Transport	3	4	9	0	9	0	9		9
*DR_j_* index			165	69	192	144	204	75	222
Priority level			4	7	3	5	2	6	1

**Table 5:** Possible design requirements and improvement actions.

Category	Items
Equipment (Machine)	The quality of the containers of the RMD (Clubs)
	The tools used for packaging such as bags, etc.)
	RMD transport boxes (trucks)
Material	The quality of the disinfector product
	The quality of water used for washing
	The quality of water used for the autoclaves
Method	The condition of the soiled medical devices carriage
	The condition of the clean medical devices carried to the autoclave
	The condition of the sterile medical devices carried to the block
Environment	A location reserved for washing and conditioning
	A location reserved for sterilization
Personnel (Man)	Staff qualification for the washing activity
	Staff distribution in the washing and conditioning step
	Staff distribution in the sterilization step
Measure	Control of the box type before loading the autoclave
	Control of the box quantity loaded in the autoclave
	Control after the sterilization step
Management	Sterilization service and block coordination
	Average duration of the sterilization process
	The availability of RMDs at a given time

## Data Availability

Data are contained within the article.

## Abbreviations

CEA	Cause and Effect Analysis
*CR*	Customer Requirement
*CR_i_*	Customer Requirement of activity *i*
QFD	Quality Function Deployment
D	Detection
*DR*	Design Requirement
*DR_ij_*	Score of Design Requirement *j* for activity *i*
FMEA	Failure Modes and Effects Analysis
HOQ	House of Quality
HSP	Hospital Sterilization Process
ICAM	Integrated Computer-Aided Manufacturing
IDEF0	ICAM Definition for Function Modeling
RPN	Risk Priority Number
O	Occurrence
RMD	Reusable Medical Devices
S	Severity
SADT	Structured Analysis and Design Technique
TQM	Total Quality Management
VOC	Voice of the Customer
*W_i_*	Importance Weight of Activity *i*
